# Identification of Rat Testicular Leydig Precursor Cells by Single-Cell-RNA-Sequence Analysis

**DOI:** 10.3389/fcell.2022.805249

**Published:** 2022-02-15

**Authors:** Xiaoju Guan, Panpan Chen, Minpeng Ji, Xin Wen, Dan Chen, Xingyi Zhao, Fu Huang, Jiexia Wang, Jingjing Shao, Jiajia Xie, Xingxing Zhao, Fenfen Chen, Jing Tian, Han Lin, Barry R. Zirkin, Ping Duan, Zhijian Su, Haolin Chen

**Affiliations:** ^1^ Zhejiang Provincial Key Laboratory of Anesthesiology, Department of Anesthesiology, The Second Affiliated Hospital and Yuying Children’s Hospital of Wenzhou Medical University, Wenzhou, China; ^2^ Department of Gynecology and Obstetrics, The Second Affiliated Hospital and Yuying Children’s Hospital of Wenzhou Medical University, Wenzhou, China; ^3^ Department of Pharmacology, The Second Affiliated Hospital and Yuying Children’s Hospital of Wenzhou Medical University, Wenzhou, China; ^4^ Department of Biochemistry and Molecular Biology, Johns Hopkins Bloomberg School of Public Health, Baltimore, MD, United States; ^5^ Guangdong Provincial Key Laboratory of Bioengineering Medicine, Department of Cell Biology, Jinan University, Guangzhou, China

**Keywords:** ScRNA-seq, Leydig cell, stem Leydig cell, CD90, testis, rat

## Abstract

Stem Leydig cells (SLCs) play a critical role in the development and maintenance of the adult Leydig cell (ALC) population. SLCs also are present in the adult testis. Their identification, characteristics, and regulation in the adult testis remain uncertain. Using single-cell RNA-seq, we found that the mesenchymal stromal population may be involved in ALC regeneration. Upon ALC elimination, a fraction of stromal cells begins to proliferate while a different fraction begins to differentiate to ALCs. Transcriptomic analysis identified five stromal clusters that can be classified into two major groups representing proliferation and differentiation populations. The proliferating group represents stem cells expressing high levels of CD90, Nes, Lum, Fn and Gap43. The differentiating group represents a progenitor stage that is ready to form ALCs, and specifically expresses Vtn, Rasl11a, Id1 and Egr2. The observation that the actively dividing cells after ALC loss were not those that formed ALCs suggests that stem cell proliferation and differentiation are regulated separately, and that the maintenance of the stromal stem cell pool occurs at the population level. The study also identified specific markers for the major interstitial cell groups and potential paracrine factors involved in the regulation of SLCs. Our data suggest a new theory about SLC identity, proliferation, differentiation, and regulation.

## Introduction

Leydig cells, localized in the interstitial compartment of the testis, produce testosterone (T). T is essential for the development and maintenance of male reproductive function (Nef and Parada, 2000). Reduced serum T, which often occurs in aging men but also can occur in young men, affects millions of men worldwide. Reduced serum T results from reduced Leydig cell steroidogenic function, and can cause increased body fat, fatigue, reduced bone mineral density, reduced muscle mass, sexual dysfunction, depressed mood, decreased cognitive function, and reduced immune response (McHenry Martin, 2012; Huhtaniemi, 2014). Thus, the development and maintenance of the appropriate number and function of Leydig cells is of fundamental importance.

Stem cells present in the neonatal testis give rise to the adult Leydig cells (ALCs) (Ge et al., 2006). Prior to and during puberty, the SLCs undergo proliferation and then differentiation to form the ALCs (Teerds and Huhtaniemi, 2015). Four distinct stages of ALC development have been identified: SLCs, progenitor Leydig cells (PLCs), immature Leydig cells (ILCs), and ultimately ALCs (Chen et al., 2017). It has been shown that the adult testis also contains SLCs, and that after the experimental elimination of the existing ALCs, these cells are able to proliferate and their progeny to differentiate to regenerate the ALC population (Teerds et al., 1990; Chen et al., 2010). As yet, however, the adult SLCs have not been well characterized, and there is little information about their regulation. In part, this is due to uncertainty about how to recognize the cells.

There are studies that have identified markers for SLCs, including Nestin (Davidoff et al., 2004; Jiang et al., 2014), COUP-TFII (Kilcoyne et al., 2014), ARX (Miyabayashi et al., 2013), CD51 (Zang et al., 2017), p75NTR (Zhang et al., 2017), PDGFRA (Ge et al., 2006; Landreh et al., 2013), GLI1, TCF21 (Shen et al., 2021), CD248 (Xia et al., 2020) and CD90 (Li et al., 2016). These markers have been identified in SLCs from different species (human to rodents) or developmental stages (fetal, neonatal, adult). It remains unclear whether these markers identify different developmental stages of a similar group of cells, or different cell types that are age- or species-specific. Indeed, the possibility of multiple cellular origins of Leydig cells has been suggested for the rodent testis (Kumar and DeFalco, 2018; Rotgers et al., 2018; Shima et al., 2018).

In the present study, we used single-cell RNA-sequencing (scRNA-seq) to analyze the changes in cells of the interstitial compartment of adult rat testes before and after ALCs were eliminated by ethane dimethanesulfonate (EDS) treatment. We provide evidence herein that the whole mesenchymal stromal population may be involved in ALC regeneration. Thus, ALC elimination induced a fraction of stromal cells to proliferate while a different fraction of the population begins to differentiate to ALCs at the same time. Transcriptomic analysis classified the heterogeneous stromal population into two major functional groups: a SLC-containing proliferation group that is capable of expanding the progenitor pool, and a progenitor-containing group that is able to form ALCs. The study also identified a group of potential paracrine factors derived from ALCs, blood vessels, and immune cells that may affect the stromal population. Thus the current study suggests a new theory about the identity, development and paracrine regulation of SLCs in the adult testis.

## Materials and Methods

### Animals and Treatment

Adult male Sprague Dawley rats of 90 days of age were purchased from Shanghai Animal Centre (Shanghai, China). Rats were housed in the animal facilities of the Second Affiliated Hospital of Wenzhou Medical University at 22°C, 12-h light, 12-h dark, with free access to water and rat chow. To eliminate Leydig cells from the testes, rats were injected with a single dose of EDS (i.p., 80 mg/kg BW) dissolved in a mixture of DMSO:PBS (1:3). Testes were collected from three rats per group at 0, 1 and 3 weeks after the rats received a dose of vehicle or of EDS. All Leydig cells were eliminated by 1 week post EDS, and new Leydig cells began to appear by 3 weeks. All animal procedures were reviewed and approved by the Laboratory Animal Ethics Committee of Wenzhou Medical University, and were performed in accordance with the Guide for the Care and Use of Laboratory Animals of NIH (NIH publication #85-23, revised in 1985).

### EdU Label of Dividing Cells

To label the dividing pool of SLCs, six rats were treated with one dose of EDS (80 mg/kg BW) and then with EdU (i.p., 50 mg/kg BW) on days 2, 4 and 6 after EDS. Testes were collected from three rats at each of 1 and 3 weeks after EDS treatment, fixed in paraformaldehyde (4%, 6 h), and embedded in paraffin. Dividing cells were identified by the Click-iT procedure. Sections were stained for CYP11A1 to identify Leydig cells. The tissues were then co-stained with the DNA dye DAPI. Percentages of EdU+/CYP11A1-, EdU-/CYP11A1+, and EdU+/CYP11A1+ cells were determined by counting the cells of about 20 fields (six to eight fields per rat). The counting fields were about 1,600 μm^2^ (400 μm × 400 μm).

### Preparation of Interstitial Cell Suspensions

To eliminate possible contamination from blood cells, the testicular artery was cannulated and perfused with DMEM/F12 culture medium containing 2.2 g/L Hepes, 0.1% BSA, 0.7 g/L sodium bicarbonate, pH 7.4). Interstitial tissue and seminiferous tubules of the testes were then separated with fine forceps under a transillumination dissection microscope (Mäkelä et al., 2020). Interstitial tissue from the testes of each of three animals was combined, and then digested with 1 mg/ml collagenase-IV in DMEM/F12 medium at 34C for 30 min with slow shaking (90 cycles/min). After allowing the undigested tissue to settle, the dispersed cells were filtered through a 30 µm pore nylon mesh. Cell viability, assayed by 0.4% Trypan blue staining, was above 85% for cells from controls and from rats after EDS.

### Single-Cell RNA-Sequencing (scRNA-Seq)

10× Genomics’ single-cell RNA-seq (scRNA-seq), involving analysis of transcriptomes on a cell-by-cell basis through the use of microfluidic partitioning to capture single cells and prepare next-generation sequencing cDNA libraries, was done by Novogene (Beijing, China). About 20,000 cells were loaded onto chromium micro-fluidic chips with 3′v2 chemistry and barcoded to achieve a targeted cell count of 10,000 with a 10× Chromium Controller. After cDNA synthesis, 14 amplification cycles were carried out for library preparations. The resultant libraries were sequenced using 2 × 150 paired-end sequencing protocol on an Illumina NovaSeq 6000 platform, with a read length of 26 bp for cell barcode and unique molecule identifier (UMI) (read 1), 8 bp i7 index read (sample barcode), and 98 bp for actual RNA read (read 2).

### Data Processing, Alignment and Clustering Analysis

For the purpose of quality control, we used FastQC to perform basic statistics on the quality of the raw reads. The FASTQ files were analyzed with the Cell Ranger Software Suite (version 2.2; 10× Genomics). Demultiplexed raw sequencing reads were processed and aligned to Rat Genome NCBI Rnor6.0 (ftp://ftp.ncbi.nlm.nih.gov/genomes/all/GCF_000001895.5_Rnor_6.0/) by 10× Genomics Cell Ranger (v3.0.0) pipeline to generate the filtered gene-barcode matrices. Cell Ranger uses an aligner called STAR, which performs splicing-aware alignment of reads to the genome. Cell Ranger then uses the transcript annotation GTF to bucket the reads into exonic, intronic, and intergenic, and by whether the reads align (confidently) to the genome. If the read is compatible with a single gene annotation, it is considered uniquely (confidently) mapped to the transcriptome. Only the reads that were confidently mapped to the transcriptome were used for UMI counting. For each gene and each cell barcode, UMIs were counted to construct digital expression matrices. A gene with expressions in more than three cells was considered as expressed, and each cell was required to express at least 200 genes to be counted. Datasets from different samples were integrated using Cell Ranger aggr (10× Genomics). Principal component analysis was run on the normalized gene-barcode matrix for the clustering analysis. The Loupe Cell Browser v5.0.0 (10× Genomics) was used to visualize results.

To further eliminate the low quality cells and potential doublets, the cells past the above FastQC procedure were projected by Loupe Browser (v5.0). The cells that co-expressed all the major markers of any two major cell types, usually the tiny clusters between the major clusters, were eliminated due to the high possibility of doublets. Also, a large cluster that contained cells with the lowest UMI counting, high mitochondrial gene reading, and no clear marker genes detected was also eliminated due to the possibility that there were no viable cells. With those quality control procedures, a total of 33,500 high quality cells were selected for further analysis.

### PCA and tSNE Analysis

In order to reduce the gene expression matrix to its most important features, Cell Ranger uses Principal Components Analysis (PCA) to change the dimensionality of the dataset from cells × genes to cells × M where M is a user-selectable number of principal components. For visualizing data in 2D space, Cell Ranger passes the PCA-reduced data into t-SNE (t-Stochastic Neighbor Embedding), a nonlinear dimensionality reduction method.

### Quality Controls for Biological and Technical Replicates

To avoid possible biological differences in the cell preparations among the individual animals, we pooled cells from three animals for cell capture and sequencing. As a way to gauge technique reproducibility, we compared the Pearson correlations for all, differentially expressed, and non-differentially expressed genes. The differentially expressed genes (DEGs) refer to the genes whose average expression levels in the cells within each of the three individual groups are found to differ statistically between any two of the three comparing pairs (*p* < 0.05), regardless of the cell types. This procedure is based on the assumption that if the differences among the individual samples are derived from biological variations, removing the biological variables, DEGs, should significantly reduce the overall variabilities among the individual samples. However, if technique variables play a major role, the differences should distribute across the whole transcriptomes. Removing a few DEGs should not significantly affect the overall variables (Pearson correlation co-efficiency).

### Cell Type Identification and Marker Exploration

To define the cellular types in detail, we projected the data into t-SNE by Loupe Browser. Through up-setting K-means from 2 to 10 stepwise, the major clusters for germ cells, immune cells, and non-immune somatic cells were identified. With graph-based clustering, more detailed cell types were defined with the help of published markers for testicular somatic cell populations. Upon defining the detailed cell types, the potential markers were identified by the “up-regulated genes per cluster” feature table. The distributions of the top five genes were checked manually by Loupe Browser and used for heatmap plot.

### Analysis of EDS Effects on Interstitial Cell Numbers and Leydig Cell Heterogeneity

With the help of Loupe Browser, the effect of EDS treatment on the major interstitial cell types were analyzed. To further analyze Leydig cells, they were identified by their expression of Cyp17a1. To examine whether any of the Leydig cells of control animals or newly formed cells in 3 weeks EDS-treated animals might have fetal Leydig cell characteristics, the top 200 enriched genes of the three groups were compared to published mouse (Sararols et al., 2021) and human (Lottrup et al., 2017) marker genes for fetal and adult Leydig cells, and the numbers of shared gene were calculated.

### Enrichment Analysis of Marker Genes

Gene Ontology (GO) enrichment analysis of marker genes was implemented by the clusterProfiler R package, in which gene length bias was corrected. GO terms with corrected *p*-value less than 0.05 were considered significantly enriched by marker gene. The top 10 terms for each Biological_process, Cellular_component, and Molecular_function were shown.

### Pseudotime Trajectory Analysis

Leydig cells and the clusters that have the potential to contribute Leydig cell precursors (mesenchymal and blood vascular cells) were used to construct pseudotime trajectories. The pseudotime was generated by Monocle based on highly variable genes identified by Seurat. All heatmaps were generated in pseudotime order, and line plots were plotted in pseudotime order with fitted curves.

### Analysis of Gene Expressions Among the Populations With Specific Markers

To compare the differences and similarities among the subclusters of mesenchymal stromal populations, the cells from the two extreme clusters (6 and 2) were pulled out and their transcriptomes were compared with committed Leydig cells (cluster 9). To further examine the relationships among the reported SLC markers Pdgfra, Nr2f2, Arx, p75ntr, Cd51, Nes, Gli1, and Cd90, the positive cells for each of the markers were pulled out from the subclusters and their transcriptomic correlations were calculated.

Detailed information about the reagents and software used are listed in Supplemental Table S1.

### Statistical Analyses

Data are expressed as the mean ± standard error of the mean (SEM). For comparisons of two groups, a Student t test was used. For comparisons of multiple groups, one-way analysis of variance (ANOVA) was applied. If group differences were revealed by ANOVA (*p* < 0.05), differences between individual groups were determined with the SNK test, using Sigma Stat software (Systat Software Inc., Richmond, CA). Values were considered significant at *p* < 0.05.

## Results

### Cellular Heterogeneity in the Interstitial Tissue of the Adult Rat Testis

**FIGURE 1:**
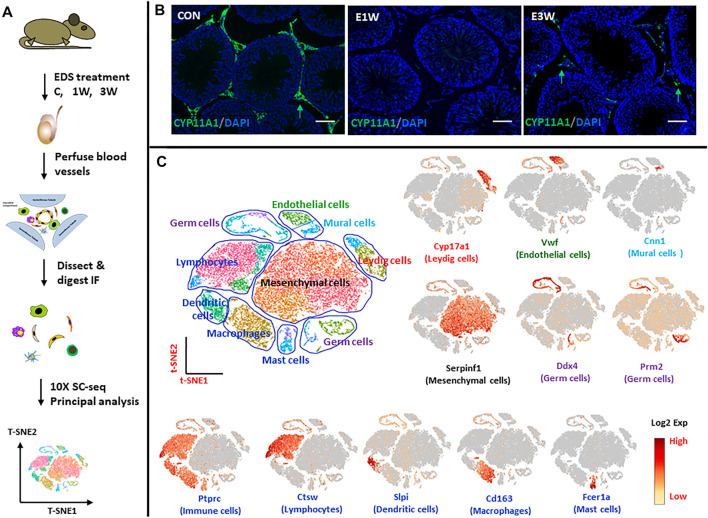
scRNA-seq analysis of rat testicular interstitial cells before and after EDS treatment. **(A)** Schematic illustration of the experimental workflow. **(B)** Immuofluorescence staining of CYP11A1 showing the disappearance and reappearance of Leydig cells (green arrows) after EDS treatment. **(C)** tSNE and clustering analysis of single-cell transcriptome of the interstitial cells of control (CON; *n* = 11,408), 1 week (E1W; *n* = 7,433) and 3 weeks (E3W; *n* = 14,659) after EDS treatment. Each dot represents a single cell and is colored according to its origin, cell type or expression level (Log2 Exp). Typical markers for each cell type are also shown. Nine major cell types were identified. tSNE, t-distributed stochastic neighbor embedding. Bars represent 50 μm in length.

With the objective to identify and characterize the SLCs in the adult testis, we analyzed cells of Sprague Dawley rat interstitial tissue by scRNA-seq (×10 Genomics) (Figure 1A). Control rats and rats at 1 and 3 weeks after receiving EDS were used for these studies. As seen in Figure 1B, Leydig cells, identified in the interstitial compartment of testis sections by their staining for CYP11A1, were eliminated from the testes by 1 week after treatment of the rats with EDS, and partially restored by week 3. This ALC depletion and early stage of regeneration enabled us to identify the SLCs that may proliferate by week 1 and progenitors that differentiate by week 3.

Cells were obtained from interstitial tissue separated from the seminiferous tubules of testes of the three groups of rats (EDS-C, EDS-1wk, EDS-3wk). This study captured 11,408, 7,433 and 14,659 cells from the interstitial tissue of control rats and rats at 1 week and 3 weeks post-EDS, respectively, with average numbers of genes of 1,226, 1,293 and 1,254 detected for the three samples, respectively. The cells from the three samples were combined and analyzed by Seurat software. Data were visualized by t-Stochastic Neighbor Embedding (t-SNE). With graph-based classification, 25 cell clusters were seen (Figure 1C). Using well established gene markers to identify specific cells of the interstitial compartment, these 25 clusters were found to contain nine distinguishable cell types. The cell types (number of clusters, typical marker gene) are: Leydig cells (two clusters, Cyp17a1), mesenchymal stromal cells (five clusters, Serpinf1), vascular endothelial cells (one cluster, Vwf), vascular mural (one cluster, Cnn1), dendritic cells (two clusters, Slpi), macrophages (two clusters, Cd68), lymphocytes (four clusters, Ctsw), mast cells (two clusters, Fcer1a), and contaminant germ cells (six clusters, Ddx4 and Prm2).

**FIGURE 2:**
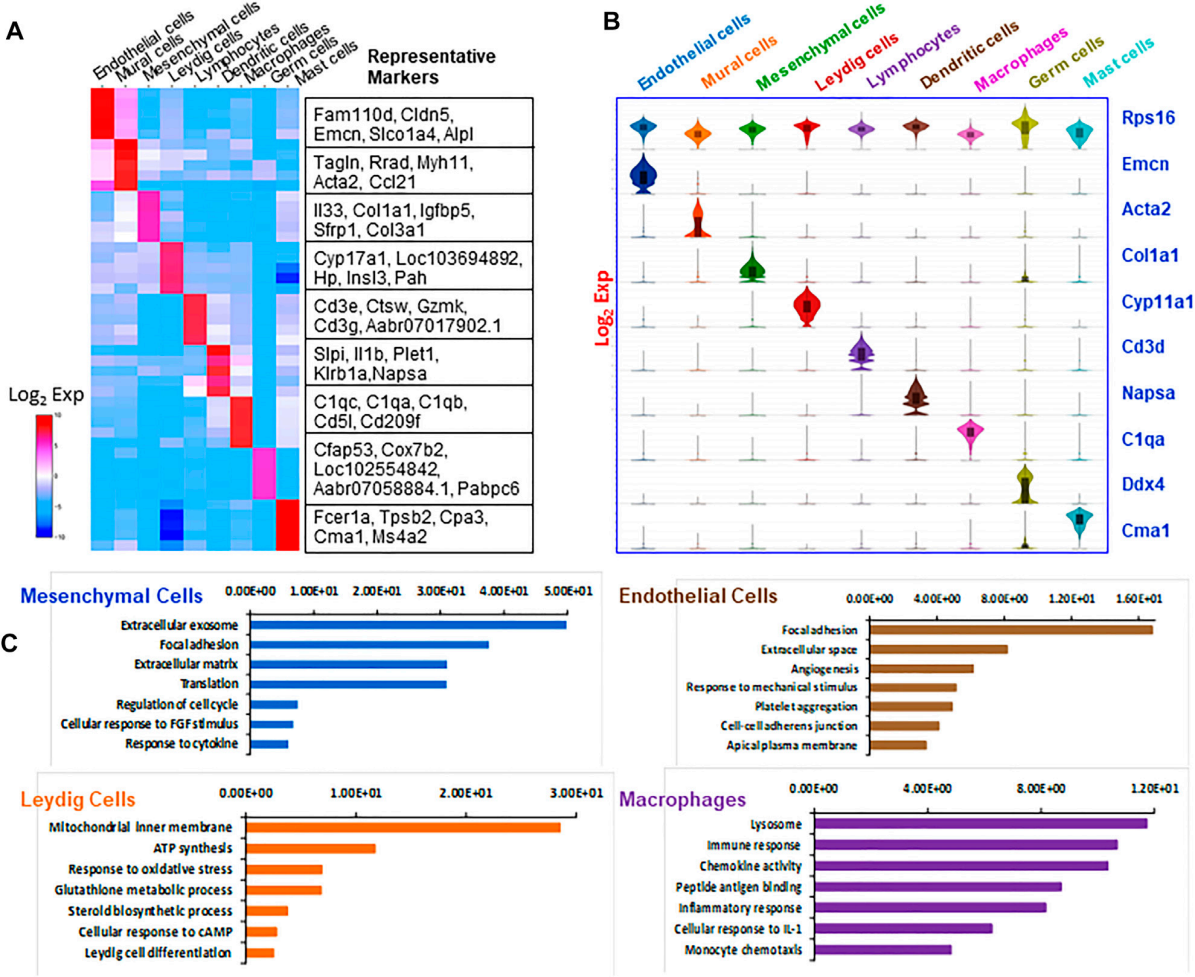
Overview of markers and attributes of the testicular cell types identified by scRNA-seq. **(A)** Heatmap shows the top markers associated with each of the major cell types identified. **(B)** Violin-distributions of the marker genes exclusively expressed by each cell type identified. **(C)** The top seven gene ontology (GO) terms enriched for the four major cell types.

To characterize the nine cell types further, the top five differentially expressed genes by each were visualized by heat map (Figure 2A), and the specificity for the key marker genes for each cell type was displayed by Violin plot (Figure 2B). The ribosomal gene Rps16, which was expressed universally across all the cell types, was used as a positive control. To further confirm the accuracy of cell identification, the top 200 enriched genes for each cell type were compared to the marker genes identified by a previous study of mouse testicular cells (Green et al., 2018). We found that most genes matched between the two studies for each cell type (Supplemental Table S2), confirming the consistency in cell identification between the two studies. As the current study does not include seminiferous tubule-associated somatic cells, the high match found between our mural cells and myoid cells as reported by Green et al. (2018) support the conclusion that the two cell types share similar characteristics.

In addition to comparing individual genes among different cell types, we compared the enriched GO terms (Figure 2C). The top enriched GO terms for four major cell types are: mesenchymal cells (extracellular exosome, focal adhesion, extracellular matrix); Leydig cells (mitochondrial inner membrane, ATP synthesis, response to oxidative stress, steroid biosynthetic process); vascular endothelial cells (focal adhesion, extracellular space, angiogenesis, response to mechanic stimulus); and macrophages (lysosome, immune response, chemokine activity, peptide antigen binding and inflammatory response). These enriched GO biological processes are consistent with the major biological functions of each cell type, confirming the accuracy of the cell identifications. As our study focused on the Leydig cell lineage, further analyses of immune cells and contaminating germ cells were not carried out.

### Effect of EDS Treatment on Interstitial Cell Populations

**FIGURE 3:**
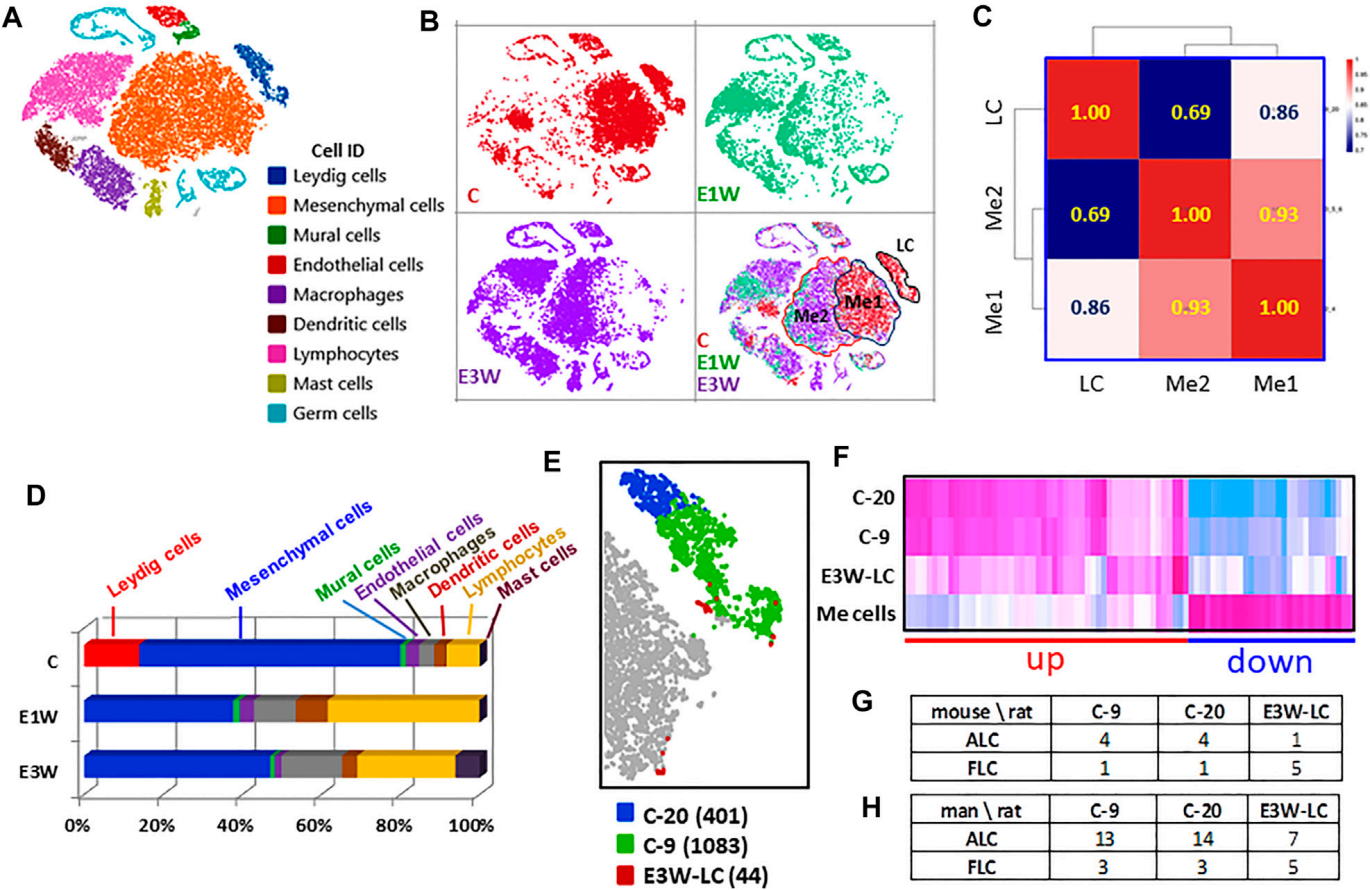
Effects of EDS treatment on the major interstitial cells. **(A)** Nine testicular cells identified. **(B)** t-SNE distributions of the three groups of cells. **(C)** Pearson correlations among mesenchymal subcluster 1 (Me1) and subcluster 2 (Me2) and Leydig cell (LC). **(D)** Effect of EDS treatment on cell numbers. **(E)** Three sub-clusters of Leydig cells, including cluster 20 (C-20, 401 cells), cluster 9 (C-9, 1,083 cells) of CON and newly formed Leydig cells of E3W (E3W-LC, 44 cells). **(F)** Heatmap of the 3 Leydig cell clusters and combined mesenchymal cluster (Me) cells. **(G,H)** Number of DEGs shared by three Leydig cell clusters with published fetal and adult Leydig cell marker genes.

With the establishment of cell identities (Figure 3A), we analyzed the effect of EDS treatment on these populations. Splitting the cells from three samples showed clear differences among them, especially between the control (CON) and two EDS groups (Figure 3B). To rule out variability caused by technique error, we calculated Pearson correlations for the total, DEGs, and no-DEGs among the three samples (Supplementary Figure S1). The correlation of the total gene list was high among the three samples (r = 0.90 and above), though the correlation between the two EDS samples was much higher (r = 0.98) than correlation with CON sample (r = 0.90). However, the differences between the CON and the two EDS groups were lost when the 603 DEGs were removed. The correlation coefficients for the 18,962 no-DEG genes were equal to 0.99, suggesting that the technique differences among the three samples are low.

In addition to eliminating Leydig cell clusters, EDS treatment also affected the mesenchymal population (Figure 3B). The disappearance of mesenchymal subcluster 1 (Me1) could be due either to cell loss or to changes in transcriptomes so that the cells moved to a different location (mesenchymal cell subcluster 2, Me2; Figure 3B). To compare the differences between these two populations and their relationships to Leydig cells, Me1 and Me2 cells in control were compared (Figure 3C). The Pearson correlations show that the two mesenchymal subclusters are much closer (r = 0.93) to each other than to Leydig cells, and that Me1 (r = 0.86) is closer to Leydig cells than Me2 (r = 0.69).

GO enrichment of the genes up-regulated in Me1 cells includes cell interaction with extracellular matrix, increase in Tgfb signaling, and inhibition of Fgf signaling (Supplementary Figure S2). Me2 cells, however, significantly changed their transcriptional activities with increases in cell migration, cell proliferation, and cell differentiation, as well as inflammatory response (Supplementary Figure S2). These results indicate that loss of Leydig cells by EDS treatment in some way mobilized mesenchymal cells by increasing cell migration and proliferation.

We then compared cell numbers among the three samples (Figure 3D). With the loss of the whole Leydig cell population, there were significant increases in immune cell populations in two EDS samples. These included macrophages, dendritic cells, lymphocytes and mast cells. The latter only appeared by 3 weeks after EDS. The increase in immune cells was dramatic such that the percentage of the mesenchymal population decreased significantly after EDS treatment. However, since the procedure to enrich the interstitial cells may itself create bias, these numbers may not represent actual cell ratios *in vivo*.

### Leydig Cell Heterogeneity and Their Relationships to Fetal and Adult Leyidg Cell Characteristics

With graphic clustering, Leydig cells in the control group were split into two sub-populations (clusters-9 and -20, Figure 1C). Due to the low recovery of the newly formed Leydig cells in the E3W group, there were only 44 Leydig cells detected in the group (Figure 3E). We combined these cells into a new Leydig cell cluster (E3W-LC) and compared this cluster with the two adult Leydig cell sub-clusters (C-9, C-20) and mesenchymal cells (Me cells). The heatmap of the DEGs among the four groups is shown in Figure 3F. Though the adult Leydig cells were clustered into two sub-groups (C-9 and C-20), they showed little difference in the DEGs. However, the E3W-LC group showed a significant difference from both the C-9 and C-20 groups. Compared to the Me cells, there were genes that were either up-regulated (red line) or down-regulated (blue line) in both the C-9 and C-20 groups (Figure 3F). In these two combined gene groups, the E3W-LC group was in the middle of the Leydig cell and Me cells, confirming that E3W-LC is transcriptomically between the precursors and the well-developed Leydig cells.

There are reports that Leydig cell heterogeneity may exist in the adult testis (Yokoyama et al., 2019), including the presence of fetal Leydig cells (Shima, et al., 2015). To examine whether any of the three Leydig cell sub-groups detected in the current study have fetal Leydig cell characteristics, the top 200 enriched genes of the three groups were compared to published mouse (Sararols et al., 2021) and human (Lottrup et al., 2017) fetal and adult Leydig cell markers. The number of genes of the two control clusters (C-9 and C-20) matched to both fetal and adult Leydig cells were remarkably similar to both mouse and human cells (Figures 3G,H). However, the newly formed Leydig cells (E3W-LC) matched fewer ALC genes and more FLC genes than the two control clusters, suggesting that E3W-LCs are closer to FLCs than C-9 and C-20. Since the matched gene numbers were relatively low, we consider the data to neither support nor disprove current theories about the presence of FLCs in the adult testis.

### Cell Marker Identification of Interstitial Leydig-Related Cell Types

The specific markers for the four Leydig cell-related populations (Leydig, mesenchymal, vascular endothelial, and mural cells) are summarized in Supplementary Figure S3. There were a total of 72 genes exclusively expressed by Leydig cells, the majority of which were not related to steroidogenesis. Additionally, we identified 53 genes specific to mesenchymal stromal cells, 48 genes for vascular endothelial cells, and 13 genes for vascular mural cells. Ace2, a key gene that mediates the invasion of Coronavirus-19 into target cells, was exclusively expressed by vascular endothelial and mural cells, suggesting that these two cell types may serve as potential targets of the virus in the testis. Regarding ALCs, most previously identified markers are related to the steroidogenic pathway. In the current study, we found that ALCs also specifically express genes with no known steroidogenic function, including Aldh1a1, Ass1, Atp2b3, Chrna4, Cyp2a1, Cyp2e1, Cyp4f4, Fads1, Folr1, Gsta4, Gsta5, Hao2, Hp, Hpx, Kcnk3, Mgst1, Pah, Prlr, Spint3, Susd3, Tst, and Ugt1a5. The functions of these genes include detoxification, redox regulation, glutathione metabolism, vesicle-mediated transport, and innate immune modification.

Among the genes specifically expressed by mesenchymal cells are those that belong to three major functional groups: extracellular matrix (Col1a1, Col3a1, Col5a1, Fbn1, Fn1, Ecm2, Efemp1, Tnfaip6, Wfdc1); immune and inflammation-related (C4b, Ccl11, Ccl20, Il1rl1, Il33, Clec3b, Clec4f, Ptgs2); and ligand/signaling-related (Fgf7, Fgf10, Fstl1, Fzd2, Gli1, Gli2, Igfbp5, Igfbp6, Inhba, Ptch2, Rtn4r, Spon1, Vegfd, Wnt4). These classifications imply that the mesenchymal stromal cells in the testis have diverse functions, including immune modification. For vascular endothelial cells, the marker genes can be classified into the following groups: transporting molecules across cell membranes (Abcg2, Ace2, Prom1,Slc1a1, Slc9a3r2, Slco1a4); angiogenesis (Cd144, Cdh5, Flt1); Notch related (Dlk1, Dll1, Dll4, Jag2); immune function and chemotaxis (Ecscr, Esm1, Mal, Sele, Selp); focal adhesion (Emcn, Itga2b, Itga6, Mmrn2, Podxl, Vwf); and signaling/transcription (Pde9a, Pdgfb, Tek, Tnfrsf19, Tnfrsf26, Erg, Foxf1, Sox7, Sox15, Sox17). The major functional processes for vascular mural cells are cytoskeletal and signaling-related (Acta2, Actg2, Cnn1, Col12a1, Mustn1, Myh11, Tagln, Mmrn1), suggesting the smooth muscle nature of the cells.

### Stem Leydig Cell Identification: Pseudotime Analysis of Vascular and Mesenchymal Cell Populations

**FIGURE 4:**
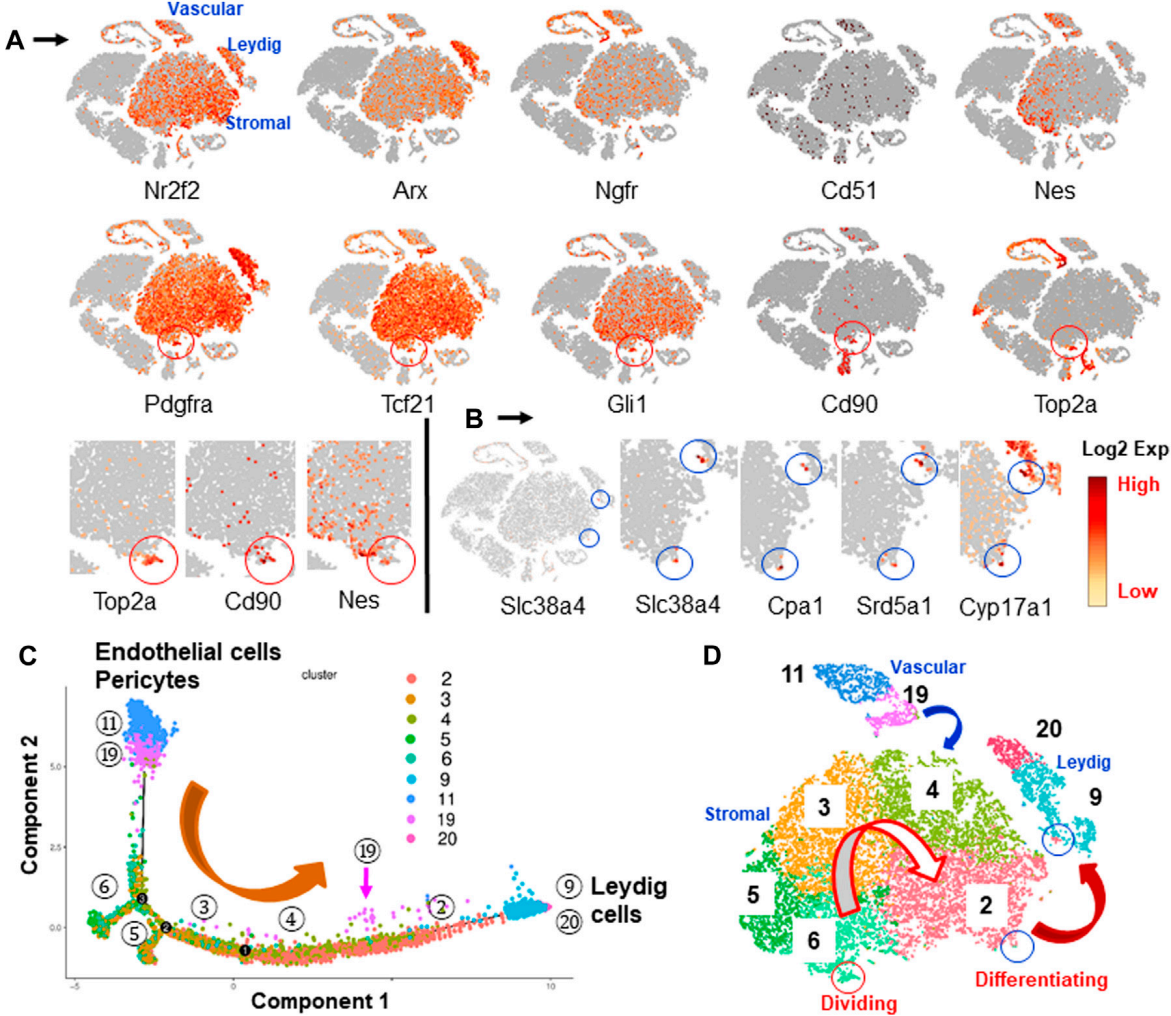
Identification and characterization of potential SLCs in the interstitial cells. **(A)** Distributions of nine SLC markers reported previously by various researchers. Cd90 and Nes concentrated in the area containing dividing cells (Top2a, red circle). **(B)** PLC/ILC markers (Slc38a4, Cpa1 and Srd5a1) and LC marker (Cyp17a1) highlighted two spots across the mesenchymal and Leydig cell clusters (blue circle), suggesting the developmental bridges between the two cell types. **(C)** Pseudotime trajectory of vascular and mesenchymal cell populations shows the developmental sequence if they were involved in LC formation. **(D)** Developmental relationships among the potential LC precursors summarized from marker genes and pseudotime trajectory.

In order to identify the potential SLC clusters, we applied previously reported SLC markers to the data, including Pdgfra, Nr2f2, Arx, Ngfr, Cd51, Tcf21, Gli1, Cd248, Nes and Cd90. All but Cd248 were detected. These markers labeled a common mesenchymal population (Figure 4A). Among the genes, Nr2f2, Arx and Pdgfra were broadly expressed by Leydig cells and mesenchymal cells. The other genes were not expressed by ALCs, but instead were expressed by mesenchymal and blood vascular, mast or germ cells. Nes and Cd90 showed some degree of selectivity in labeling the large central mesenchymal island (Figure 4A).

Since elimination of ALCs by EDS triggers SLC proliferation within the first week (Teerds et al., 1990; Yan et al., 2000; Chen et al., 2010), the observation that mesenchymal cells gained dividing activity after EDS treatment suggests that these cells might represent true SLCs. Interestingly, the specific dividing genes, including Top2a and another 8, co-localized to the same area as Nes+ and Cd90+ cells in the mesenchymal “island” (red circles, Figure 4A; Supplementary Figure S4). Among the nine SLC marker genes, only 5 (Pdgfra, Gli1, Tcf21, Nes and Cd90) significantly labeled the Top2a+ dividing fraction. At higher magnification, it is clear that although both Nes and CD90 were concentrated in the dividing area, not all dividing cells expressed Nes (Figure 4A). These results indicated that among all nine reported marker genes, Cd90 is the only one that showed selectivity in identifying a specific cluster within the large mesenchymal population, and also co-localized with dividing cells. However, unlike other markers, Cd90 was also expressed by newly invaded mast cells by week 3 after EDS treatment.

To further analyze the relationships among the mesenchymal sub-populations and the newly formed Leydig cells, the genes co-expressed by mesenchymal and Leydig cell populations were determined. Three genes, Slc38a4, Cpa1 and Srd5a1, were found to be expressed exclusively by small numbers of cells (blue circles) crossing the edges of mesenchymal and Leydig cell clusters (2 and 9) (Figure 4B; Supplementary Figures S5,S7). Interestingly, these two spots were also consistently labeled by ALC markers (Cyp17a1 and Loc103694892), suggesting that the cells in these two spots represent an intermediate state that bridges the mesenchymal (cluster 2) and Leydig (cluster 9) cell populations. Also, among the five sub-clusters, only cluster 2 has cells shared with Leydig cell cluster (9) (Supplementary Figure S7).

Pseudotime trajectory analysis is a proven way to determine the developmental relationships among differentiating lineages. The tool calculates the relationships among the cell populations based on specific transcriptomic signatures. We have analyzed the relationships among Leydig cells (clusters 9 and 20) and their potential precursors (mesenchymal clusters two to six and vascular clusters 11 and 19) (Figure 4C; Supplementary Figures S8, S9). The vascular endothelial (cluster 11) and mural (cluster 19) clusters were included since the vascular cells were reported to be important precursors of Leydig cells in the adult rat testis (Davidoff, 2019; Davidoff et al., 2004, 2009). The trajectory arranged vascular cells on one end of the branches while the two Leydig cell populations (clusters 9 and 20) were arranged on the other end (Figure 4C). Interestingly, all five mesenchymal clusters (2–6) were arranged sequentially in between, with cluster two being the closest to and cluster 6 the farthest from the Leydig cell population. Also, some of the mural cells (19 with pink arrows) were arranged along the way (between 4 and 2) to LCs, suggesting their potential to contribute immediate ALC precursors.

**FIGURE 5:**
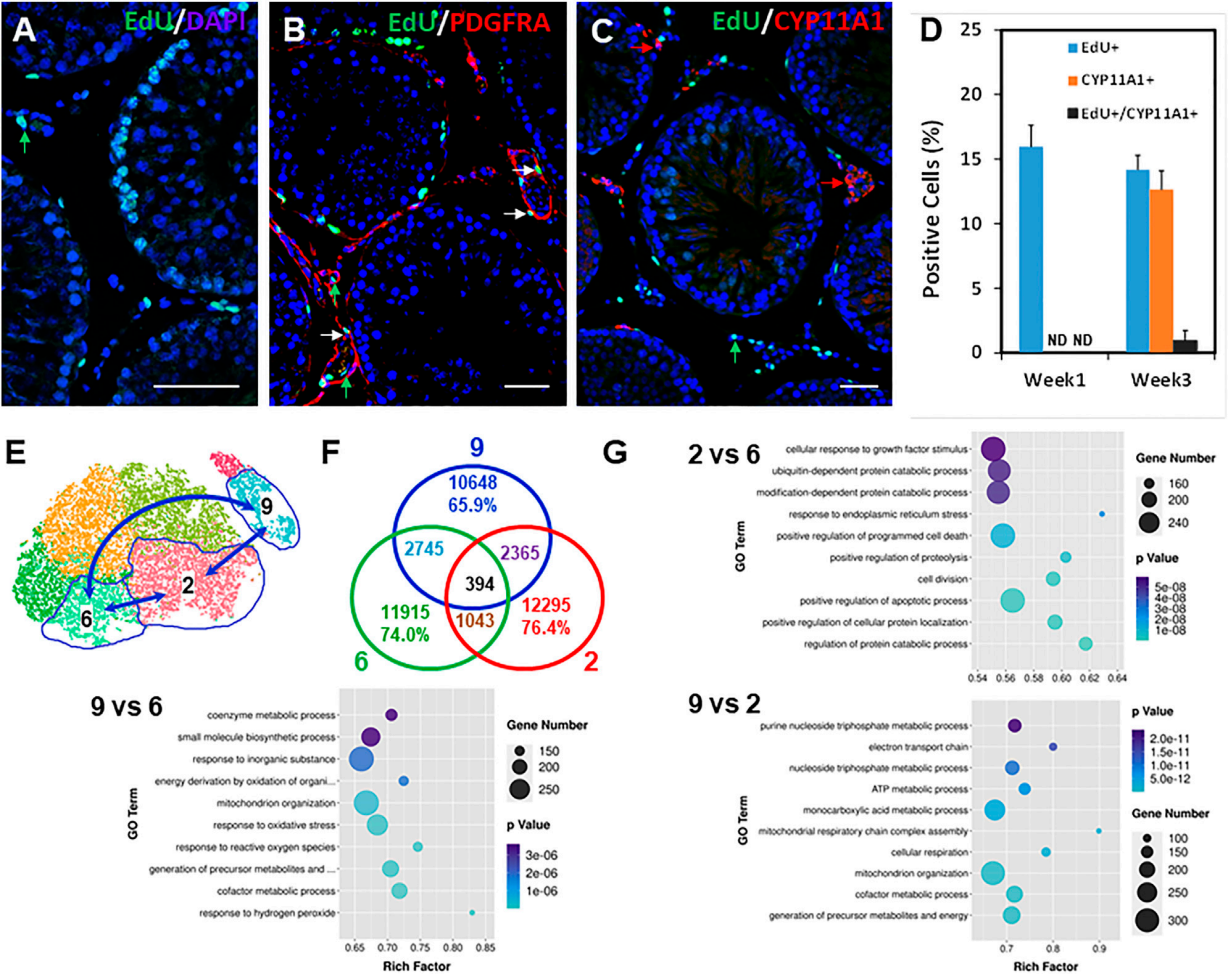
Relationship between dividing SLCs to LC development and GO analysis of potential SLC clusters. **(A)** EdU labeling of the dividing interstitial cell pool during the first week after EDS. **(B)** Co-staining the EdU+ (green) and PDGFRA+ (red) cells 1 week after EDS treatment. **(C)** Co-staining the EdU+ (green) and CYP11A1+ (red) cells 3 weeks after EDS treatment. **(D)** Quantification of the EdU+ (green) and CYP11A1+ cells from three animals (mean ± SE). **(E)** Cell clusters selected for transcriptomic comparison. **(F)** Differentially expressed genes (DEGs) shared by three comparing pairs. **(G)** Top 10 GO terms enriched for the three comparing pairs. *p* values for all the enriched GO terms are less than 6 × 10^−8^. Green arrows: EdU-only positive nucleus; Red arrows: PDGFRA- or CYP11A1-only positive cells; White arrows: EdU/PDGFRA or EdU/CYP11A1 co-stained cells. Bars represent 50 μm in length.

Trajectory analysis suggests a potential developmental sequence from vascular cells to mesenchymal cells and further to Leydig cells (Figure 4D). The model implies that all the cells in the central mesenchymal clusters have the potential to form Leydig cells, but with different abilities to do so depending on the locations of the cells (from 6 to 2). This conclusion is also supported by the distributions of nine dividing markers (Figure 4A; Supplementary Figure S4) and the early committed progenitor Leydig cells (Slc38a4+, Cpa1+ and Srd5a1+) (Figure 4B; Supplementary Figures S5–S7). Specifically, the dividing cells contained in cluster 6 located furthest from Leydig cells, while the newly committed Leydig cell-containing cluster 2 is the immediate population to form Leydig cells (Figure 4D). Cluster 6 needs to develop through clusters 5, 3, and then four to eventually reach to the edge of population 2. This model suggests that cells forming Leydig cells are not the ones newly generated by divisions after EDS. To confirm these hypotheses, we labeled the dividing interstitial cell pool by repeated EdU administration to rats on days 2, 4 and 6 after EDS treatment, and then assessed the percentage of the newly formed Leydig cells (CYP11A1+) that also were EdU+ by week 3 (Figure 5C). With three EdU injections, numerous EdU+ cells were found in the interstitial compartment by day 7 after EDS treatment, but CYP11A1+ containing cells were not present (Figure 5A). Co-staining of the sections with PDGFRA antibody revealed that most of the dividing cells were also PDGFRA positive, confirming that the dividing cells after EDS treatment are potential SLCs (Figure 5B). Interestingly, some of the PDGFRA-negative cells were also labelled after EdU treatment (green arrows, Figure 5B). These cells appear to be endothelial cells of blood vessels. By 3 weeks, however, both CYP11A1+ and EdU+ cells were visible in the interstitial compartment, but very few, if any, co-expressed the two markers (Figure 5C). This was confirmed by quantification of EdU+ and CYP11A1+ cells at weeks 1 and 3 post-EDS (Figure 5D). The results support the hypothesis that the proliferative (Top2a+) cells after ALC loss may represent a true stem population that must undergo progenitor transition steps in order to form ALCs. The cells forming Leydig cells immediately after EDS treatment, therefore, were not the actively dividing stem cells in cluster 6, but rather the cells already present in the testis (cluster 2).

### Transcriptomic Analysis of Mesenchymal Cell Subpopulations

The five mesenchymal sub-clusters may represent different developmental stages of progenitors in forming ALCs, and therefore may differ in their ability to divide and differentiate. We wished to determine how their transcriptomes might differ. To do so, we compared Leydig cells (cluster 9) and the two key mesenchymal stages, namely the initial stage containing the dividing “stem” population (cluster 6), and the final stage containing newly differentiating Leydig cells (cluster 2) (Figure 5E). Among a total of about 16k transcripts detected for each of the three groups, 1,043, 2,365, and 2,745 transcripts were differentially expressed in clusters 2 vs 6, 9 vs 2, and 9 vs 6 as determined by comparing pairs with ratios >1.5 and *p* < 0.05 (Figure 5F). The number of DEGs between groups 6 and 2 (1,043) was fewer than between 2 and 9 (2,365) and 6 and 9 (2,745), confirming that the transition from mesenchymal (clusters 6 and 2) to Leydig cells (cluster 9) represents a major developmental leap compared to the minor changes within the mesenchymal population itself (6 vs 2). Additionally, the exclusive DEGs between cluster pairs of 6 vs 9 (2,745) is more than that of 2 vs 9 (2,365), supporting that cluster 6 is developmentally farther away from cluster 9 than cluster 2 (Figure 5F).

The volcano distributions of the significant genes are shown in Supplementary Figure S10. The 100 most significant DEGs are listed in Supplementary Tables S3–S5. The 30 most significantly enriched GO terms are shown in Figure 5G and Supplementary Figure S10. Among the most significantly enriched terms in the cluster pairs are: 2/6, protein catabolic process, ER stress, apoptosis, growth stimulation, mitochondrial function, and cell division and adhesion; 2/9, cellular respiration and mitochondrial function, cofactor metabolism, NADH, and redox regulation with no down-regulated function detected in the top 30 terms; and 6/9, response to oxidative stress, mitochondrial organization and function, cell adhesion, cofactor metabolism, NADH and redox regulation. The last group was very similar to the pair of 2/9, suggesting that the major changes from mesenchymal to Leydig cells were shared by clusters 2 and 6.

### New Markers for Mesenchymal Cell Subpopulations

**FIGURE 6:**
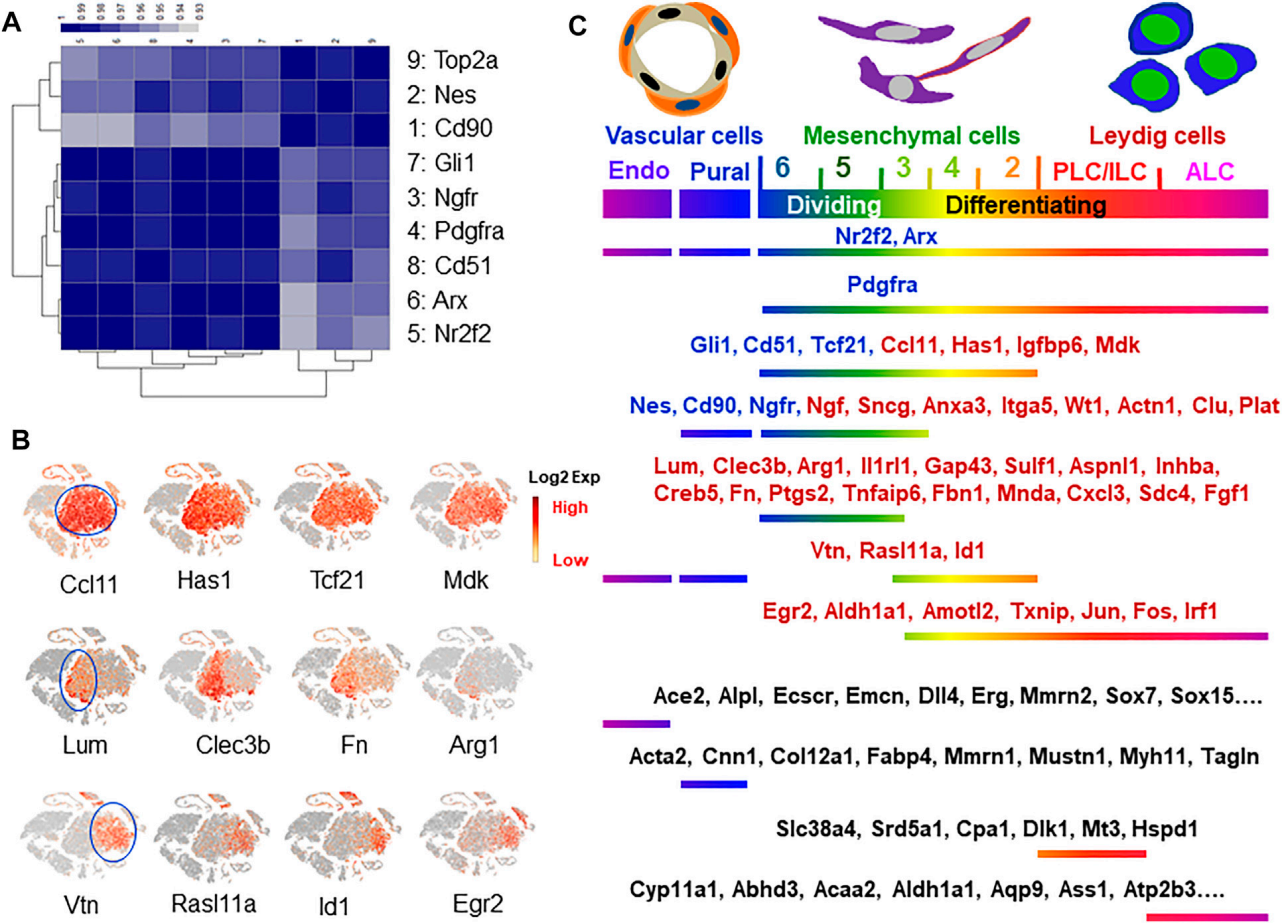
Marker gene identification and characterization. **(A)** Correlational coefficient among the eight reported SLC markers and dividing marker Top2a. **(B)** New marker genes identified with even or specific expressions concentrated on either left-side (clusters 4–6) or right-side (clusters 2–3) of mesenchymal cell “island”. **(C)** Distributions of the reported and newly identified markers for cells related to Leydig cells.

Among the previously reported nine SLC marker genes, only Nes and Cd90 labeled a fraction of cells in the whole mesenchymal population (Figure 4A). To further analyze the relationships among the cells expressing each of the 9 marker genes and the dividing gene (Top2a+), we pulled the positive cells for each of the 9 markers from the whole mesenchymal population and compared their transcriptomes. The correlation efficiencies of the transcriptomes among the nine groups are summarized in Figure 6A. The color represents the correlation efficiency between two of the populations involved. The nine cell populations can be classified into two major groups. The larger dark square on the low-left corner indicates that 6 markers, Gli1, Ngfr, Pdgfra, Cd51, Arx and Nr2f2, had higher correlation-efficiency and therefore closer relationships. The remaining 3 markers, Top2a, Nes and Cd90, however, are combined into a smaller, deep-colored square on the up-right corner, indicating a closer relationship among the three. This result is consistent with the above observations that the 6 markers labeled cells evenly across the whole mesenchymal population, while the 3 (Nes, Cd90 and Top2a) labeled a special group of cells within cluster 6. Overall, the results support the conclusion that Nes and Cd90 may be better markers for true SLC than the others since they labeled cells within the dividing population after ALC loss.

To find new specific markers for each of the dividing-associated clusters (5+6) and the differentiating-associated clusters (2+4), we systematically analyzed the distributions of the mesenchymal cell-specific genes. The majority (60%; 52/86 genes) showed evenly expressing patterns across all five sub-clusters, while 28 and 12% preferentially labeled each of the dividing (5+6, left-side) or the differentiating clusters (2+4, right-side), respectively (Figure 6B). Among the new markers identified, Ccl1, Has1, Igfbp6 and Mdk labeled the whole mesenchymal population without selectivity. These genes identify the whole mesenchymal populations as Pdgfra, Nr2f2 and Arx do (Figure 4A), but with better specificity since they do not label side-clusters such as ALCs and vascular cells at significant levels. Similarly, Lum, Clec3b, Fn, and Inhba clusters associated with dividing populations (clusters 5, 6), as did Nes and Cd90 (Figure 4A). In contrast to Nes and Cd90, these genes were not expressed by vascular cells or mast cells. Interestingly, relatively fewer genes were found with a preference for labeling the differentiating clusters 2+4. Among the representatives of this category are Vtn, Rasl11a, Id1 and Egr2 (Figure 6B). However, none of these is perfect since they also label the side-clusters such as vascular cells or ALCs. More of the specific genes for these three categories can be found in Supplementary Tables S3–S5 and Supplementary Figure S11. The distributions of the eight reported SLC marker genes (blue font) and the newly identified genes (red font) are summarized in Figure 6C. The specific genes for vascular and differentiating Leydig cells were also included (black font). More of these marker genes can be found in Supplementary Figure S3.

### Potential Paracrine Regulatory Factors for Mesenchymal Cell Populations

**FIGURE 7:**
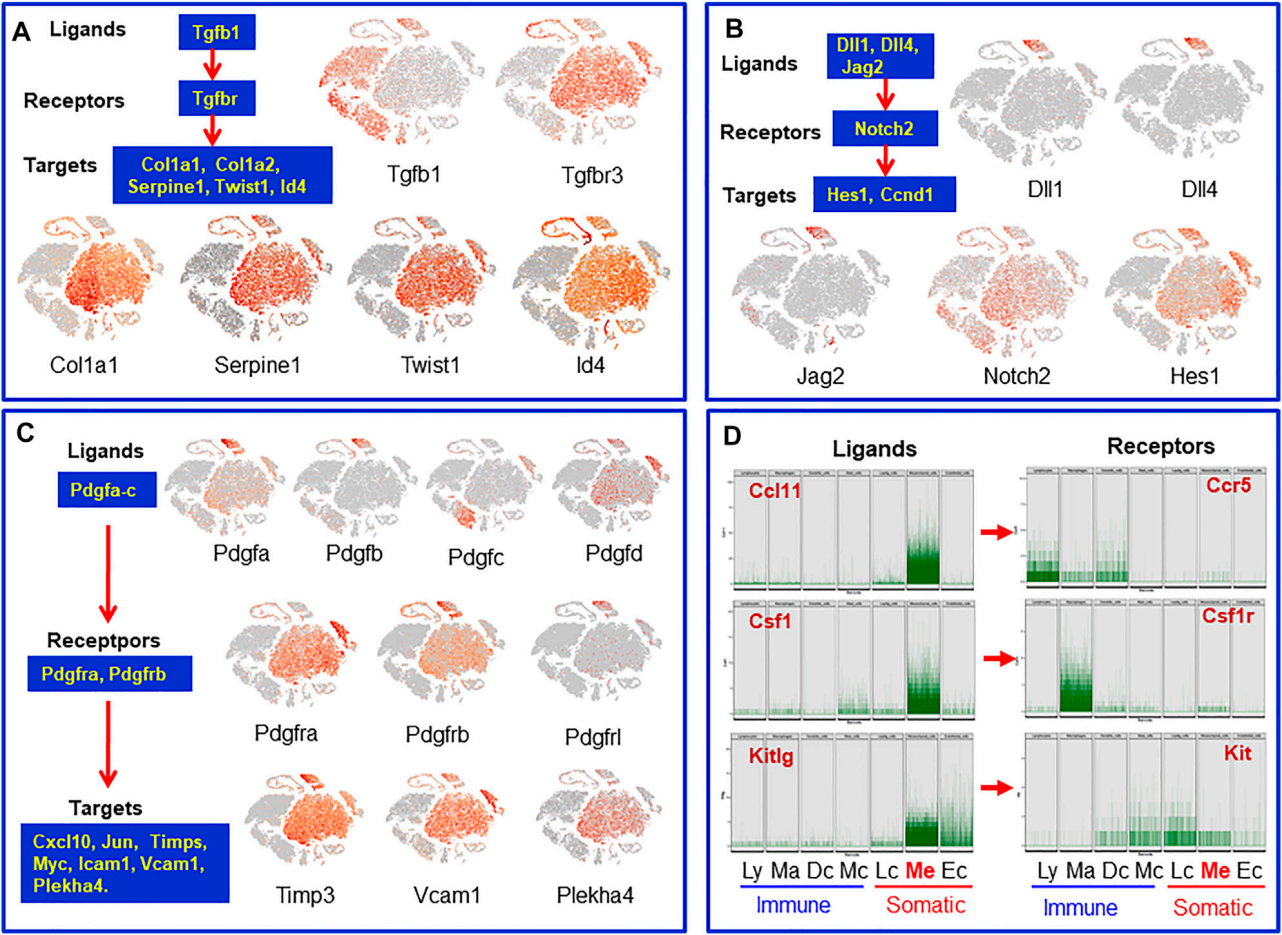
Cells and their paracrine regulatory factors that have potential to target mesenchymal SLCs or be targeted by factors from mesenchymal SLCs. **(A)** Tgfb family. **(B)** Notch family. **(C)** Pdgf family. **(D)** Transcript levels of individual cells of the mesenchymal cluster whose protein receptor genes were expressed by neighbor interstitial cells. Ly, lymphocytes; Ma: macrophages; Dc, dendritic cells; Mc, mast cells; Lc, Leydig cells; Me, mesenchymal cells; Ec, endothelial cells.

Tissue stem cell functions are regulated by niche factors. To identify the potential local regulatory factors for SLCs, we analyzed receptor transcripts of mesenchymal cells and transcripts for ligands expressed by the neighboring interstitial cells. Tgfbr3 and the target genes Col1a1, Id4 and others were found to be expressed by mesenchymal cells while the Tgfbr ligand Tgfb1 is specifically expressed by vascular endothelial cells and the immune cells (Figure 7A). Similarly, vascular endothelial cells were found to express the Notch ligands Dll1/4 and Jag2, while mesenchymal cells express Notch2 (receptor) and its target gene Hes1 (Figure 7B). It is well known that the Pdgf family plays a critical role in the development of testicular interstitial cells (Basciani et al., 2010). However, the ligands and receptors involved are not well established. We found that Pdgfa and Pdgfb are almost exclusively expressed by vascular cells, while Pdgfc and Pdgfd are primarily expressed by macrophages and Leydig/mesenchymal cells (Figure 7C). The major receptors are expressed by mesenchymal (Pdgfra, Pdgfrb), Leydig (Pdgfra) and vascular (Pdgfrb, Pdgfrl) cells. Their target genes Timp3, Vcam1 and Plekha4 are also expressed by these three cell types. The unique expressions of the ligands and their receptors indicate that the regulatory networks of the PDGF family are far more complex than anticipated (Basciani et al., 2010). One of the surprising observations is that macrophages could be the major contributors of Pdgfc, a ligand primarily relating to Pdgfra.

**FIGURE 8:**
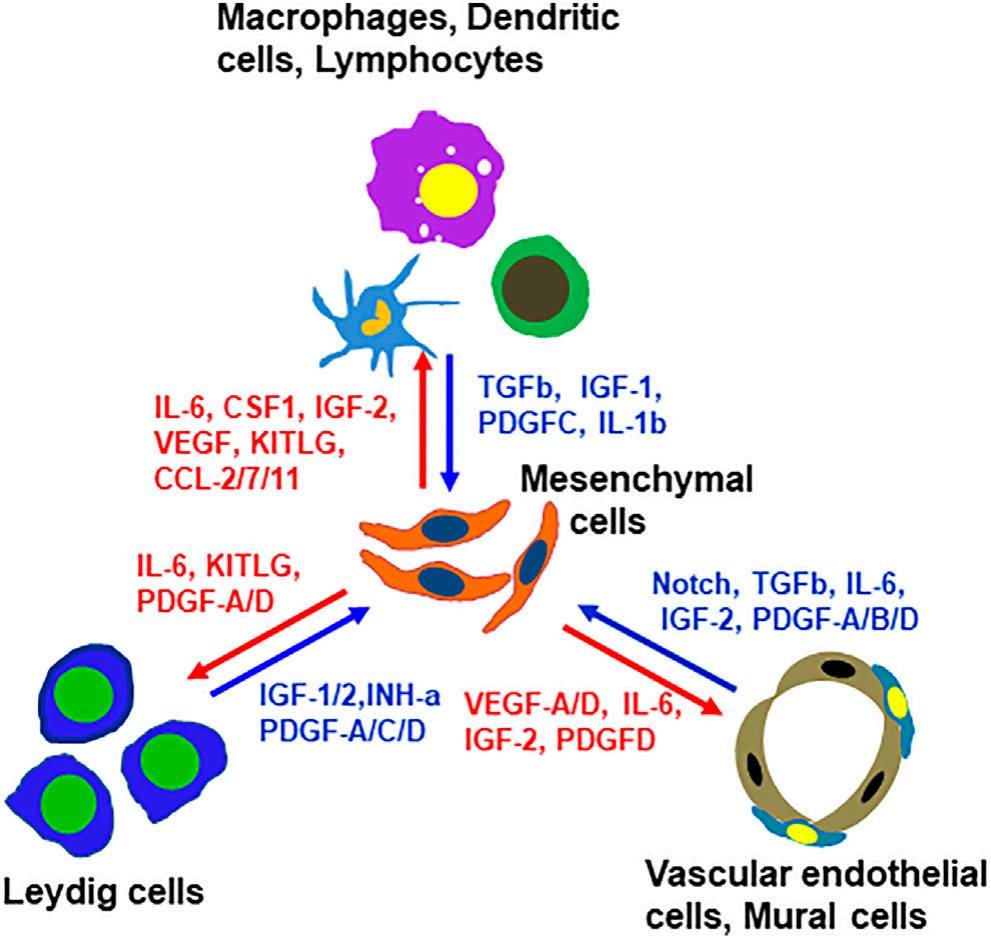
Summary of paracrine factors and their producing cells that have potential to target mesenchymal SLCs or their rounding cells. These factors were selected based on the fact that the ligands are primarily expressed by non-mesenchymal interstitial cell types and their receptors were primarily expressed by mesenchymal cells, or vice versa.

In addition to the ligands that may target mesenchymal cells, there are also mesenchymal cell-derived factors that have the potential to affect the neighboring cells. For example, mesenchymal cells may function as a primary source for C-C Motif chemokine ligands Ccl-2, -7, -11, Il6, Il33, Csf1 and Kitlg (Figure 7D and Supplementary Figure S12). These ligands may primarily or exclusively target immune cells and/or other somatic cells (Leydig cells, vascular cells or mesenchymal cells themselves) since these cells express the receptors (Figure 7D and Supplementary Figure S12). Vegfa and Vegfd are primary mesenchymal cell factors that can target endothelial, dendritic and germ cells (Supplementary Figure S12). Another interesting observation is the Igf family (Supplementary Figure S12). Mesenchymal cells are potential major sources for both Igf1 and Igf2. Also, mesenchymal cells are the major population that express the receptor and multiple Igfbps (Igfbp2-7), suggesting the importance of the family for these cells. The major factors that may be involved in the interactions of mesenchymal cells and other interstitial cells are summarized in Figure 8.

## Discussion

It has been known for some time that after the elimination of ALCs by the administration of EDS, a new generation of ALCs is established. This suggested the possibility of the presence of stem cells in the adult testis (Chen et al., 2010). Indeed, previous studies showed that cells located on the surfaces of seminiferous tubules and blood vessels are stem cells capable of giving rise to ALCs (Davidoff et al., 2004; Li et al., 2016). Whether these cells represent a specific SLC population for ALC formation, or are among the cells capable of giving rise to ALCs, had not been addressed previously. In the current study, we used scRNA-seq to identify and further characterize adult SLCs by addressing the transcriptional activity of rat testicular interstitial cells from control testes and from testes 1 and 3 weeks post-EDS. We sought to identify cell clusters that begin to divide by 1 week post EDS and subsequently differentiate to Leydig cells by 3 weeks post EDS. Based on the distributions of the nine previously reported stem cell markers (Chen et al., 2017; Eliveld et al., 2020), we found, unexpectedly, that eight were detected, but that in the interstitial cells that we studied, none was specific enough to identify the mesenchymal population exclusively without labelling side-clusters. The best among the markers was Cd90, which labeled a small fraction of the mesenchymal population that overlapped with the dividing cells induced by ALC loss. However, this fraction was not able to form ALCs readily, but rather needed to undergo intermediate progenitor transitions before forming ALCs.

### Cellular Heterogeneity in the Interstitial Compartment of Adult Rat Testis

With Graph-based classification of the interstitial cells by Loupe Browser, 25 clusters were revealed representing nine unique cell types. These included: Leydig cells (two clusters), mesenchymal cells (five clusters), vascular endothelial cells (one cluster), vascular mural cells (one cluster), macrophages (two clusters), dendritic cells (two clusters), lymphocytes (four clusters), mast cells (two clusters), and germ cells (six clusters). These multiple-cluster cell classifications suggest that there are degrees of cell heterogeneity within each of the cell types. Also, there were multiple immune cell populations in addition to macrophages identified by the current study that had not been recognized before. Further study is needed to address the possible involvement of these immune cells in such processes as orchitis, steroidogenesis and spermatogenesis.

### Cell Marker Identification for Interstitial Non-Immune Cell Types

Among the four non-immune cell types (Leydig, mesenchymal, vascular endothelial, and mural), we identified 72, 53, 48 and 13 genes, respectively, that were exclusively expressed by these cells. We found that in addition to expressing genes with steroidogenic function, Leydig cells express genes with no known steroidogenic functions including those expected to be involved with receptors (Lhcgr, Chrna4, Folr1, Gnrhr, Prlr), detoxification (Aldh1a1, Ass1, Cyp2a1, Cyp2e1, Cyp4f4, Pah, Ugt1a5), redox regulation and glutathione metabolism (Fads1, Gsta4, Gsta5, Hao2, Mgst1), and vesicle-mediated transport and innate immune modification (Hp, Hpx, Tst, Kcnk3). These results indicate that Leydig cell function extends beyond androgen production, and the receptor expressions suggest that their functions may be regulated by multiple local and systemic factors in addition to LH.

Another interesting finding of the current study relates to ALC-specific genes. In contrast to previous studies that used isolated cells to identify genes highly but not exclusively expressed by ALCs (Chen et al., 2004; Stanley et al., 2011), ScRNA-seq enabled the identification of 72 genes uniquely expressed by the ALCs. These results were consistent with those of a previous study in which Leydig-specific genes were identified by whole testicular mRNA profiles (O’Shaughnessy et al., 2014).

In addition to ALC specific genes, the current study also identified six genes (Slc38a4, Srd5a1, Cpa1, Dlk1, Mt3, Hspd1) specifically and transiently expressed by newly committed PLCs and/or ILCs. The genes were briefly turned on during the SLC to ALC transition. The expressions of these genes by a small group of cells across mesenchymal and Leydig clusters represent a developmental bridge that connects the two populations, consistent with the relationship between the two cell types as defined by pseudotime analysis. Although the biological significance of these genes is largely unknown, they can serve to specifically identify the intermediate cell stages between SLCs and ALCs.

Among the genes specifically expressed by mesenchymal cells, the majority fall into three major functional groups: extracellular matrix (Col1a1, Col3a1, Col5a1, Fbn1, Fn1, Ecm2, Efemp1, Tnfaip6, Wfdc1), immune and inflammation-related (C4b, Ccl11, Ccl20, Il1rl1, Il33, Clec3b, Clec4f, Ptgs2), and growth factors and signaling (Fgf7, Fgf10, Fstl1, Fzd2, Gli1, Gli2, Igfbp5, Igfbp6, Inhba, Ptch2, Rtn4r, Spon1, Vegfd, Wnt4). The expressions of these genes suggest that the interstitial mesenchymal cells play important roles in extracellular matrix maintenance, growth factor production, immune modulation, and anti-inflammation.

The genes specifically expressed by vascular endothelial cells can be classified into six functional groups: transporting molecules across cell membranes (Abcg2, Ace2, Prom1,Slc1a1, Slc9a3r2, Slco1a4), angiogenesis (Cd144, Cdh5, Flt1), Notch signaling (Dlk1, Dll1, Dll4, Jag2), immune and chemotaxis (Ecscr, Esm1, Mal, Sele, Selp), focal adhesion (Emcn, Itga2b, Itga6, Mmrn2, Podxl, Vwf), and signaling and transcriptional factors (Pde9a, Pdgfb, Tek, Tnfrsf19, Tnfrsf26, Erg, Foxf1, Sox7, Sox15, Sox17). The major functional groups for mural cells are smooth muscular cytoskeletal and signaling (Acta2, Actg2, Cnn1, Col12a1, Mustn1, Myh11, Tagln, Mmrn1), and mesenchymal stem cell functions (Fabp4 and Map3k7cl). These cells share a number of marker genes with the myoid cells identified by Green et al. (2018) and others, suggesting the similarity of the two cell types. These results suggest that vascular cells not only provide support for the vascular system, but also may be involved in the regulation of testicular function by producing paracrine factors.

### Mesenchymal Cells as Candidates for Leydig Cell Generation

The most unexpected findings of the current study are the recognition that the cells capable of forming Leydig cells may belong to the large mesenchymal population, and that only a small fraction of that population may serve as a true stem population capable of actively dividing and the progeny differentiating upon ALC loss. These conclusions are based on the following observations:

First, none of the nine published SLC marker genes is expressed exclusively by any one cluster, though all nine were expressed commonly by the mesenchymal population. Seven of the marker genes (Pdgfra, Nr2f2, Arx, Dcf21, Ngfr, Cd51 and Gli1) were expressed in cells across the whole mesenchymal population, and only 2 (Nes and Cd90) were expressed focally, but still within the large population. Cd90 correlated best with the division marker Top2a. The correlation coefficients of the transcriptomes among the cells expressing the 9 markers were all above 0.94, indicating their high similarities. We did find genes with preferred relationships to either dividing (6+5+3) or differentiating (2+4) clusters. The dividing clusters are further away from Leydig cells and therefore are more likely to contain cells with stem characteristics. The genes associated with this group included Lum, Clec3b, Arg1, Il1rl1, Gap43, Sulf1, Aspnl1, Creb5, Fn, Ptgs2, Tnfaip6, Fbn1, Mnda, Cxcl3 and Sdc4; and those expressed by the differentiating clusters included Vtn, Rasl11a and Id1. Others in the latter group (Egr2, Aldh1a1, Amotl2, Txnip, Jun, Fos and Irf1) were also co-expressed by ALCs.

Second, pseudotime analysis indicated that the five mesenchymal subclusters may all be involved in Leydig cell regeneration. Cells associated with cluster 6 were defined as the most undifferentiated group. These cells need to transit through clusters 5, 3, 4 and eventually to 2 in order to finally form Leydig cells. This sequential developmental model is supported by cell division and differentiation activities. Among the five mesenchymal subclusters, only cluster 6 contained dividing cells after EDS, indicating its “stem” nature to expand the whole mesenchymal pool. This is consistent with pseudotime results which defined cluster 6 as a developmental starting point. On the other hand, the three PLC markers Slc38a4, Cpa1 and Srd5a1 that highlight the transition from SLCs to ALCs consistently labeled two small cell fractions across mesenchymal cluster 2 and Leydig cluster 9, confirming that cluster 2 represents the mesenchymal fraction mostly ready to form Leydig cells.

Third, transcriptome comparisons among mesenchymal clusters 6 and 2 and Leydig cluster 9 provide similar conclusions. Among the 16,000 transcripts detected for each of the 3 clusters, the number of specific DEGs between clusters 6 and 2 (1,043) was significantly fewer than these found between clusters 2 and 9 (2,365) and 6 and 9 (2,745), confirming that the differences among both mesenchymal sub-populations (6 and 2) and Leydig cells (cluster 9) are far more significant than the difference within the two mesenchymal sub-populations, 6 and 2. However, DEGs between clusters 6 and 9 (2,745) are more than that between two and 9 (2,365), supporting the conclusion that cluster 6 is farther away than cluster 2 from Leydig cluster 9.

Fourth, and perhaps most importantly, we found that very few, if any, of the newly differentiated Leydig cells came from the early dividing mesenchymal cells. It is well-established that in EDS-treated adult rats, the most active cell proliferation in the testicular interstitium occurs within the first week (Teerds et al., 1990; Yan et al., 2000; Chen et al., 2010). To label the whole dividing interstitial cell pool, we injected three doses of EdU within the first week after EDS treatment. After 3 weeks, we quantified the number of newly formed Leydig cells and found very few that contained EdU co-staining. These results indicate that cells forming Leydig cells are not the dividing cells following EDS treatment.

Taken together, these four lines of evidence support the conclusion that precursor cells forming Leydig cells belong to the general mesenchymal population. A specific Cd90-expressing fraction of the mesenchymal population was reserved as true stem cells that can expand the whole mesenchymal cell pool by actively dividing upon ALC loss. Also, the vascular mural cells can function as the Leydig precursors. The newly formed mesenchymal and mural cells need progenitor transitions in order for the cells to be able to form ALCs. This implies that both proliferation and differentiation are regulated at the population level.

### Paracrine Factors for the Mesenchymal Stromal Cells

To address the potential paracrine factors for SLCs, we focused on the ligands expressed by the neighboring interstitial cells, and the receptors and/or target genes expressed by the mesenchymal cell population. Based on these criteria, we identified multiple ligands from Leydig, vascular and immune cells with potential targets on mesenchymal cells. These include Pdgf, Notch, Dhh, Bmp4/7, Tgfb, Fgf12, Igf1/2, Ngf, Il-1b, Il-4 and Il-6. These results indicate that the functions of the interstitial mesenchymal cell population are regulated by multiple factors from diverse cell types.

One of the unexpected findings of the current study relates to the PDGF family. Sertoli and Leydig cells were long considered the major sources for the Pdgf ligands in the testis (Basciani et al., 2010). However, our results indicate that vascular endothelial cells are the major, if not the only, cell type that produce both Pdgfa and Pdgfb, while macrophages are the primary cell type for Pdgfc. Additionally, Leydig/mesenchymal cells contribute to Pdgfd production. The targets of these Pdgf ligands include mesenchymal/Leydig cells (Pdgfra) and mesenchymal/vascular cells (Pdgfrb). The unique expressions of the Pdgf ligands and their receptors by different interstitial cells revealed a far more complex regulatory network among the interstitial cells than previously recognized for this family.

## Data Availability

The datasets presented in this study can be found in online repositories. The names of the repository/repositories and accession number(s) can be found below: NGDC Genome Sequence Archive, CRA004958; NGDC BioProject, PRJCA006440.
