# Supplementary material for: Identification of Rat Testicular Leydig Precursor Cells by Single-Cell-RNA-Sequence Analysis
Source: Front Cell Dev Biol. 2022 Feb 15;10:805249. doi: 10.3389/fcell.2022.805249 (PMC8887666; doi:10.3389/fcell.2022.805249)
Supplement: Supplementary file 1 [file DataSheet1.docx]

**Supplementary Materials for**

**Guan et al., “Identification of Rat Testicular Leydig Precursor Cells by Single Cell RNA-Seq”**

**Supplemental Table S1**

REAGENT OR RESOURCE SOURCE IDENTIFIER

**Antibodies**

Rabbit Anti-CYP11A1 Cell Signaling Technology Cat# 14217S

Rabbit Anti-PDGFRA Abcam Cat# Ab203491

Goat Anti-Rabbit IgG H&L Abcam Cat# Ab150077

Goat Anti-Rabbit IgG H&L Abcam Cat# Ab150078

**Biological samples**

Rat testicular tissue Shanghai Animal Centre SD rats: 3-5m old

Shanghai, China

**Chemicals, peptides, and recombinant proteins**

Fetal Bovine Serum ATCC Cat# 302020

DMEM/F12 Medium Thermo Fisher Cat# 11330032

Phosphate Buffered Saline Fisher bioreagents Cat# BP399-500

Collagenase IV Sigma-Aldrich Cat# C5138

Trion X-100 Beyotime Cat# P0096

HBSS Hank’s balanced salt solution Thermo Fisher Cat# 14175-103

Bovine Serum Albumin (BSA) New England Biolabs Cat# B9000S

Goat serum Solarbio Cat# S L038

DAPI containing coverslip solution Beyotime Cat# P0131

Paraffin wax Sigma Cat# 327204

Ethane dimethane sulfonate (EDS) SKS Chem Cat# 4672495

EDU Shanghai yuanye Bio-Tech Co Cat# s25031

Click-it® EdU Alexa Fluor Kit Thermo-Fisher Cat# C10337

Click-it® EdU Alexa Fluor Kit Thermo-Fisher Cat# C10338

Absolute ethanol (200 Proof) Fisher Cat# BP2818-500

Paraformaldehyde (4%) Solarbio Cat# P1110

Trypan blue solution (0.4%) Thermo Fisher Scientific Cat# 15250061

Xylene Changshu Hongsheng Fine Chemical Co Cat# CY2021

**Critical commercial kits**

Chromium TM SC 3ʹ Library & Gel Bead Kit v2, 16 rxns 10x Genomics PN-120237

Chromium TM Single Cell A Chip Kit, 48 rxns 10x Genomics PN-120236

Chromium TM i7 Multiplex Kit, 96 rxns 10x Genomics PN-120262

**Deposited data**

Raw fastq NDGC/GSA https://ngdc.cncb.ac.cn/gsa/s/2QvgQU09

The processed matrix files

and DEGs lists NDGC/OMIX <http://ngdc.cncb.ac.cn/omix/preview/yRPuVbr5>

**Software and algorithms**

GraphPad PRISM <https://www.graphpad.com/> Version 8.4.3

Seurat <https://satijalab.org/seurat/> Version 3.1.4

R <https://www.r-project.org/> Version 3.6.1

dmatch <https://qzhan321.github.io/dmatch/> Version 0.1

FastQC <https://github.com/s-andrews/FastQC> Version 0.11.9

Trimmomatic-0.39 program http://www.usadellab.org/cms/index.php?page=trimmomatic NA

Cell Ranger <https://github.com/10XGenomics/cellranger> Version 2.1.1

The Loupe Cell Browser <https://www.10xgenomics.com/products/loupe-browser> V5.0.0

Monocle3 <https://cole-trapnell-lab.github.io/monocle3/docs/trajectories/>

ggplot2 <https://github.com/tidyverse/ggplot2> N/A

OmicStudio online tool <https://www.omicstudio.cn/index> N/A

**Supplemental Table S2: Marker genes shared between Green et al, 2018 (mouse) and current (rat) studies**

Green, C. D. et al. A Comprehensive Roadmap of Murine Spermatogenesis Defined by Single-Cell RNA-Seq. Dev. Cell 46, 651-667.e10, doi: 10.1016/j.devcel.2018.07.025

**Supplemental Fig S1: Pearson correlations for the total, DEGs, and no-DEGs among the 3 samples.**

The correlation of the total gene list was higher between the 2 EDS samples (r=0.98) than correlations with CON sample (r=0.90). However, the differences between the CON and the 2 EDS groups were lost when the 603 DEGs were removed, with all correlations being equal to 0.99 in NO-DEGs group.

**Supplemental Fig S2: Enriched GO terms for the comparisons of mesenchymal cell 1 and 2**

**
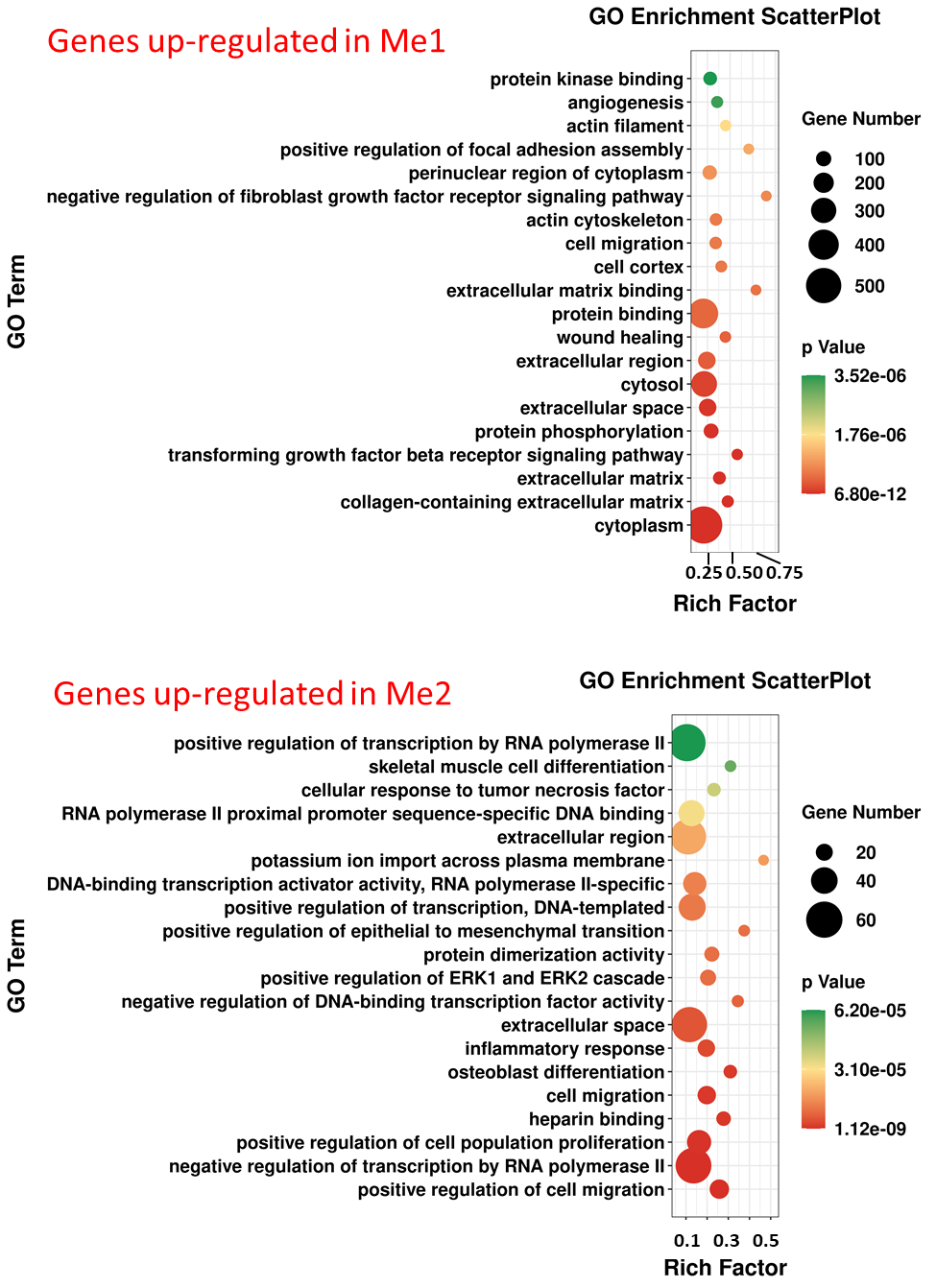
**

**Supplemental Fig S3: Marker genes for the major interstitial no-immune cell types**

The top 25 genes identified by study of O'Shaughnessy et al. (Reproduction 147, 671-682, 2014).

**Supplemental Fig S4: Expression of cell cycle genes (red circles) by mesenchymal cells**

**Supplemental Fig S5: Certain mesenchymal cell expressing some (blue circles) but not all (red circles) Leydig cell genes (low magnification)**

**Supplemental Fig S6: Expression of cell cycle genes (red circles) and Leydig cell genes (blue circles) by mesenchymal cells (high magnification)**

**Supplemental Fig S7: Cross border distribution of mesenchymal cells (cluster 2) in Leydig cell territory**

**Supplemental Fig S8: Pseudotime analysis for Leydig cell related clusters**

**Supplemental Fig S9: Pseudotime analysis for Leydig cell related clusters**

**Supplemental Fig S10: DEGs and enriched GO terms for the comparisons of clusters 6, 2 and 9**

**Supplemental Table S3: 100 most significantly regulated genes between clusters 2 and 6**

**Supplemental Table S4: 100 most significantly regulated genes between clusters 9 and 2**

**Supplemental Table S5: 100 most significantly regulated genes between clusters 6 and 9**

**Supplemental Fig S11: Genes expressed evenly or unevenly over mesenchymal population**

**Supplemental Fig S12: Transcripts whose product may be involved in the paracrine interactions of the interstitial cells.**
